# Thymidine Analogues for Tracking DNA Synthesis

**DOI:** 10.3390/molecules16097980

**Published:** 2011-09-15

**Authors:** Brenton L. Cavanagh, Tom Walker, Anwar Norazit, Adrian C.B. Meedeniya

**Affiliations:** 1 Health Institute and Eskitis Institute, Griffith University, Queensland 4107, Australia; 2 Department of Molecular Medicine, Faculty of Medicine, University of Malaya, Kuala Lumpur 50603, Malaysia

**Keywords:** DNA synthesis, EdU (5-ethynyl-2′-deoxyuridine), nucleoside analogues

## Abstract

Replicating cells undergo DNA synthesis in the highly regulated, S-phase of the cell cycle. Analogues of the pyrimidine deoxynucleoside thymidine may be inserted into replicating DNA, effectively tagging dividing cells allowing their characterisation. Tritiated thymidine, targeted using autoradiography was technically demanding and superseded by 5-bromo-2-deoxyuridine (BrdU) and related halogenated analogues, detected using antibodies. Their detection required the denaturation of DNA, often constraining the outcome of investigations. Despite these limitations BrdU alone has been used to target newly synthesised DNA in over 20,000 reviewed biomedical studies. A recent breakthrough in “tagging DNA synthesis” is the thymidine analogue 5-ethynyl-2′-deoxyuridine (EdU). The alkyne group in EdU is readily detected using a fluorescent azide probe and copper catalysis using ‘Huisgen’s reaction’ (1,3-dipolar cycloaddition or ‘click chemistry’). This rapid, two-step biolabelling approach allows the tagging and imaging of DNA within cells whilst preserving the structural and molecular integrity of the cells. The bio-orthogonal detection of EdU allows its application in more experimental assays than previously possible with other “unnatural bases”. These include physiological, anatomical and molecular biological experimentation in multiple fields including, stem cell research, cancer biology, and parasitology. The full potential of EdU and related molecules in biomedical research remains to be explored.

## 1. Introduction

Multicellular animals replicate their DNA when undergoing cell division. This occurs during embryogenesis and in the growth of tissues in the adult. DNA synthesis is the most primitive and defining event in the advent of the animal kingdom. The persistence of DNA over millions of years of animal evolution and its presence across all species alludes to its fundamental role and implies deep protective mechanisms to ensure its conservation. The four bases adenine, thymine, cytosine and guanine are the absolute unconditional coding components of DNA which is faithfully replicated during cell division, providing the robustly reproduced, blueprint for life.

DNA synthesis has a proof reading system that prevents the contamination of the bases. This tightly regulated process was initially breached using radiolabeled nucleoside analogs and subsequently with analogues detected using light microscopy. This allowed the tracking and subsequent characterization of newborn cells. During the S-phase of the cell cycle, the four DNA bases are organised by cellular machinery to form new DNA strands. The replicate paired strands are then segregated and drawn to the two poles of the mother cell prior to its cleavage to produce two daughter cells, each with a complement of newly synthesised DNA comprised of old and newly incorporated bases. If the cellular machinery is fooled into selecting and incorporating an “unnatural base” during DNA synthesis, it is possible to track the daughter cells and their offspring subsequent to that division.

## 2. A Window of Opportunity: DNA Synthesis

The duplication of the entire complement of genetic information in an organism is essential for cell division in living organisms. Initiation of DNA synthesis for cell division is highly regulated in eukaryotic cells where it is confined to the S-phase of the cell cycle [1,2]. The cell cycle, in its entirety, describes the strictly regulated and sequentially ordered processes eukaryotic cells undergo for duplication of the cell [2,3]. Order is ensured by a series of checkpoints that ensure DNA replication and the other phases of the cell cycle are executed with remarkable precision [1,3].

In eukaryotic cells, DNA is replicated by semi-conservative replication. DNA synthesis is initiated at specialized zones on chromosomes known as “origins of replication”. In S-phase these zones are bound by the origin recognition complex that serves as a dock for the binding of additional proteins necessary for initiation [1,3]. The recognition complex and associated proteins locally unwind the DNA helix, creating replication forks. Nucleic acid synthesis is initiated by the Pol α/primase complex or primosome. The primase synthesizes short RNA primers that undergo limited extension by Pol α [4,5]. These primers are bound by the DNA polymerase complex enabling DNA polymerases to initiate DNA synthesis [5,6]. The resulting RNA-DNA primers are utilized by Pol δ and Pol ε for processive elongation on the lagging and leading strands, respectively. DNA polymerases are a group of multi-enzymatic complexes that can synthesize DNA by ‘reading’ the DNA template strand and catalysing condensation reactions between hydroxyl groups of the sugar components of the nucleoside units. It is these nucleoside subunits that polymerize to form DNA [4,5,6].

Nucleosides are synthesised *de novo* within cells or diffuse into the cytoplasm. Notably, the heterogeneously distributed members of the concentrative and equilibrative nucleoside transporter families mediate active uptake of nucleosides, including thymidine [7]. They vary in their substrate specificity and together with the synthesised nucleosides, mediate cellular nucleoside homeostasis [8,9,10,11]. The nucleoside subunits provide a vehicle for a tag to be inserted into the newly synthesized DNA. Through the introduction of nucleoside analogues into cells, typically of the nucleoside base thymidine, it is possible to insert a ‘tagged’ nucleoside into newly synthesised DNA [2,3]. These ‘tagged’ thymidine molecules can subsequently be detected by several methods, principally autoradiography and immunofluorescence, and more recently by “click chemistry”.

## 3. Thymidine Analogues and Their Utility

The past few decades have seen major inroads in our understanding of the molecular mechanisms mediating DNA synthesis [1,2]. Concurrently, techniques developed by chemists are increasingly utilised in biological applications, including novel methods to study cell proliferation [1,2].

### 3.1. Radiolabeled Nucleoside Analogues

The original techniques used for the selective labelling of mitotically active cells involved ‘tagging’ thymidine using a radioactive probe and a detection step using autoradiography or scintillation techniques [3,5]. Tritium labelled (H^3^) thymidine and autoradiography was originally developed by Woods, Taylor and Hughes, as DNA probes [5]. The two assays showed that DNA replicated in a semi-conservative manner [5]. By placing crocus root tips in medium containing H^3^-thymidine it was demonstrated that the crocus roots took up tritium and that DNA, synthesized subsequently during the replication phase of the cell cycle, were tritium labelled [1,5]. When the root tips were removed from the tritium labelled media the cells observed after a second replication cycle had tritium labelling in half of the chromosomes [1,5]. This evidence substantiated the idea that newly synthesized DNA was not randomly assorted between DNA. Instead the strands had remained intact with one conserved DNA strand serving as a template for the second nascent strand. Each new single complete DNA helix was formed from an existing strand, and combined with an existing strand is termed “semi-conservative replication” [3,5].

Autoradiography provided the first evidence for two ‘neurogenic’ zones in the brains of adult mice, showing the capacity to generate new neurons into adulthood [6,12]. Kaplan and Hinds, and also Bayer, using tritium labelling and autoradiography, confirmed and extended these findings describing a rostral migratory stream consisting of migrating neuroblasts originating in the subventricular zone and extending into the olfactory bulb [6,13,14,15,16]. The use of tritium labelling and autoradiography in neurogenesis experiments has been extensive. The major drawbacks of handling radiolabelled substrate and the time consuming techniques inherent to autoradiography was the catalyst for the development of new techniques to tag nascent DNA, facilitated by advances in the production of monoclonal antibodies [2,17,18].

### 3.2. Halogen-Based Nucleoside Analogues

5-Bromodeoxyuridine (BrdU), another analogue of the nucleoside thymidine, is readily incorporated into the DNA of dividing cells during the S-phase of the cell cycle [2,17,18]. The development of an antibody specific for BrdU provided the means for immunological detection of the newly synthesized BrdU-incorporating DNA [2,17,18]. BrdU has become the method of choice for researchers experimenting on proliferation for over the past two decades. Within the biomedical sciences alone, BrdU has been employed in over 20,000 published studies. The advantages of BrdU over tritium labelling are numerous [2,17,19] including significantly reduced demand on equipment and time. BrdU enables experimentation using a variety of concurrent techniques and processes such as lineage and cell fate analysis through use of cell specific markers. Studies of cell origin and migration at high resolution together with quantitative studies of cell genesis are possible using BrdU [2,20,21,22]. The use of BrdU in studying adult neurogenesis has provided deep insights into the neuronal migratory pathway between the sub-ventricular zone and the olfactory bulb. BrdU was the key tool for substantiating the existence of adult neurogenesis in the primate and human brain [23]. BrdU has provided insight into neurodegenerative diseases, factors that influence neurogenesis, rostral migratory stream traffic and new neurons in the olfactory bulb in murine and primate models [14,24,25,26,27,28].

The halogenated thymidine analogues 5-chlorodeoxyuridine (CldU) and 5-iododeoxyuridine (IdU) mirror BrdU in targeting DNA and are also detected by means of specific antibodies [29]. These halogenated derivatives may be multiplexed to probe proliferative cells [30]. The efficacy of DNA access and labelling is generally considered to be uniform for the halogenated thymidine analogues, however detection efficiency may differ as determined by the efficacy of the antibodies targeting the unnatural bases.

The ability of nucleoside analogues to incorporate into forming strands of DNA and RNA has found application in clinical fields, specifically as anti-viral and chemotherapeutic agents. The anti-metabolic properties of nucleoside analogues are used to action a diverse range of therapeutic outcomes [31,32]. The pyrimidine analogue 5-fluoruracil was demonstrated to reduce the growth of liver tumour cells in the late 1950s [33]. Further work has shown that despite exhibiting a great deal of structural and metabolic similarity, nucleoside analogues differ in their biological effects following their incorporation into nascent DNA. These divergent effects are exemplified when considering the varied subclasses of nucleoside analogues used in the treatment of acute myeloid leukaemia, other haematological conditions and solid tumours such as colorectal and breast cancers [31,32].

BrdU and tritiated thymidine are delivered through either intracerebroventricular (i.c.v), intravenous (i.v) or intraperitoneal (i.p) injection or as an oral dose [2,12]. The nucleoside analogue molecule enters the bloodstream and permeates tissues. Once inside the tissue the chemical is available to all cells [2,12]. Dividing cells draw on the pool of nucleosides endemic to the extracellular environment [5,6]. Nucleoside analogues compete with the cell’s endogenous nucleosides for selection and incorporation into newly forming DNA [5,6]. The standardisation of dosage and dosing regime when applying nuceloside analogues remains to be resolved [12]. Many studies use a dose range of 50–100 mg/kg, however a number of studies have reported that higher doses are required to saturate the entire complement of dividing cells, as addressed later. BrdU is metabolised through a dehalogenation mechanism [2,12]. Dehalogenation of BrdU and other halogenated thymidine analogues occur via the same mechanism [12]. This mechanism acts to metabolise nucleoside analogues that have not been incorporated into newly synthesized DNA, and to clear them from the bloodstream [6]. Active metabolism of nuceloside analogues may result in the removal of a large proportion of the administered dose before it reaches target tissues [2,12].

The widespread use of BrdU to detect newly generated cells has highlighted a number of limitations in its utility. The administration of BrdU, particularly in doses above 60 mg/kg in rodents may effect cytotoxic changes in some animals and their progeny as they develop [2,34,35]. Optimal dosage for BrdU to label the entire complement of dividing cells remains uncertain. Whilst a standard dose of 50–100 mg/kg is routinely applied in rodent models, Cameron and McKay suggested that a dose of 300 mg/kg is necessary to detect the entire complement of dividing cells [35]. It is likely that physiological differences between individuals, different rodent models and different species could have effects on the bioavailability and dose-dependency of BrdU and its permeability into tissues. Comparison of the immunohistochemical localisation of the cell cycle marker Ki-67 with BrdU, showed that BrdU only labelled approximately half as many cells as Ki-67 in Wistar rats. The variability is partially explained by the specificity of BrdU for exclusively targeting the S-phase of the cell cycle [36], whilst, Ki-67 is expressed in all phases of the cell cycle, bar interphase. As such, Ki-67 detects cells that are in the cell cycle, not purely cells that have undergone S-phase DNA synthesis [36]. The benefits of using a high dose of BrdU to achieve saturation of S-phase cells, is moderated by the known toxicity of BrdU at higher doses. BrdU has also been implicated in the false labelling of cells undergoing DNA synthesis where DNA replication does not involve cell division, e.g., DNA repair and re-entry into the cell cycle as part of an apoptopic mechanism [2,35,36].

The detection of BrdU following its incorporation into DNA requires the denaturation of DNA to allow targeting by antibodies. These protocols result in cell and tissue disruption, together with the degradation of proteins and nucleic acids, limiting the utility of BrdU as a probe where concurrent measurement of protein content or molecular analysis is required. Its use as a quantitative tool for measuring rates of cell genesis and as a labelling tool for measuring cell fate is therefore limited [2,35,36]. Despite these short comings BrdU has remained the primary tool for the study of cell proliferation and cell fate.

## 4. EdU and “Click Chemistry” Light Multiple Paths

### 4.1. EdU and “Click Chemistry”

**Scheme 1:**
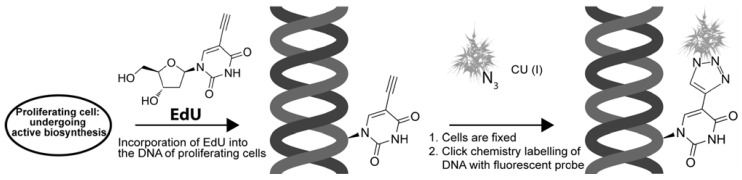
EdU and click chemistry for labeling DNA synthesis.

A new technique for detecting DNA synthesis in proliferating cells, *in vivo* and *in vitro*, has been developed in the last three years [37]. 5-Ethynyl-2’-deoxyuridine (EdU), structurally similar to the natural nucleoside, has a terminal alkyne group replacing a methyl group at the 5 position of the pyrimidine ring. EdU, which is readily incorporated into DNA during the S-phase, can be coupled via a covalent bond using “click” chemistry [37,38], namely, a copper [Cu(I)]-catalyzed [3+2] cycloaddition reaction, to a fluorescent dye-conjugated azide as described below (Scheme 1).

**Scheme 2:**
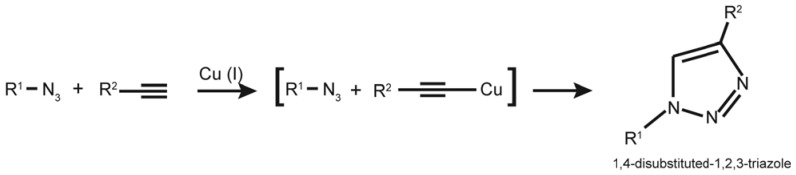
Copper-catalyzed click reaction of an alkyne and azide.

“Click” chemistry [37,38] or Huisgen’s 1,3-dipolar cycloaddition is a rapid chemical reaction that occurs readily at room temperature and is catalyzed by copper Cu(I), resulting in the formation of a covalent bond between an azide and an alkyne group as described below [39,40] (Scheme 2).

The small sized dye-azide allows for efficient EdU detection under bio-orthogonal conditions. The reaction occurs at high efficiency in a range of solvent and pH conditions, and has a narrow distribution of reactivity. Aldehyde-based fixation and detergent permeabilization enables the dye-conjugated azide (e.g., Alexa Fluor dye) access to the alkyne group of EdU. The alkyne group is un-reactive in biological systems, providing an unique opportunity for EdU to be used in tracking DNA synthesis [41,42,43].

### 4.2. BrdU *vs.* EdU

Antibody-based detection of the thymidine analogue BrdU demands DNA denaturation to facilitate steric access by antibodies [44]. This requires DNA to be denatured to form a single-strand and also the permeabilization of cell and nuclear membranes for antibody access. These harsh labelling conditions invariably degrade the specimen and introduce inconsistency to BrdU staining. Conversely, EdU can be readily detected in intact double-stranded DNA following its incorporation into DNA. The key element (the fluorescent probe used in targeting EdU), is a small, permeable molecule which readily penetrates the cell to target EdU inserted within the DNA structure [42]. The greater permeability of the fluorescent tag allows thick tissue sections and whole mount specimens to be labelled readily and reliably [45,46]. This leads to greater sensitivity of detection, together with the tissue retaining other proteins which in turn can be detected by multiplexing with standard immunofluorescence techniques [43,45,47].

#### 4.2.1. Utility in Characterising Zones of Proliferation and the “Stem Cell Niche”

**Figure 1:**
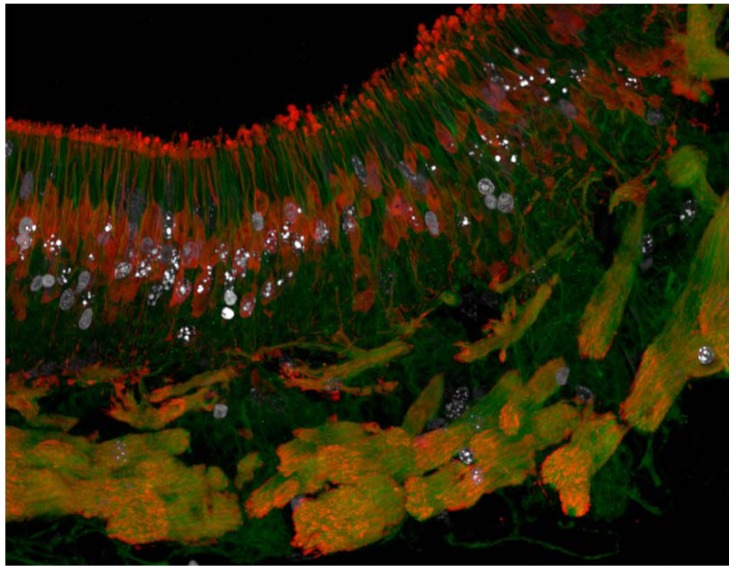
Mature (red) and immature (green) sensory neurons colocalise with EdU (white) introduced into the proliferative cells of adult rodents, one week prior to tissue harvest. The majority of cells which underwent DNA synthesis during the period of EdU exposure have differentiated into immature and mature neurons following 7 days. The fluorescence conjugated azides are commercially available. The fluorescence molecules are widely used probes of small molecular size and bright emission, ranging from the ultraviolet to infrared wavelengths. They are readily multiplexed with other fluorescence techniques including immunofluorescence. Thus, these techniques are robustly applied to track cell genesis and adult stem cells.

EdU can be used for pulse labelling and also for extended periods of cell labelling and henceforth for high resolution EdU/Hoechst quenching assays. Consequently, EdU enables highly sensitive and quantitative detection of proliferating cells and facilitates continuous cell cycle assessment [41]. In recent studies EdU was used to track cell genesis during embryonic development [48] and in adult stem cell niches [47,48]. Comparison of BrdU and EdU data allowed clear documentation of the advantages of EdU labelling as shown in Figure 1.

The “click” reaction required for the covalent bonding of the azide conjugated fluorescent probe to the alkyne incorporated into DNA, occurs rapidly. Despite the near bio-orthogonal fluorescence labelling by EdU, the preparations need to be washed following the conjugation of the azide probe to EdU, as excess probe present within the specimen produces background fluorescence. The pro-fluorogenic azide probe, 3-azido-7-hydroxycoumarin, which only emits fluorescence on the formation of the 1,2,3-triazole product was used to overcome this limitation [49]. This pro-fluorogenic compound reduces background signal as the un-reacted precursors are optically inactive. This development has application in the quantification of DNA synthesis in high throughput assays of cell proliferation.

The reversal of the typical alkyne tag and azide probe “click chemistry” to an azide tag and an alkyne probe would allow for the labelling of two distinct proliferative cell populations within the same preparation. 5-Azido-UDP (AdU) has been used to label proliferative cells [37] based on the previous work done by Sunthankar and colleagues [50]. Using an azide tag increased background florescence, as seen previously in activity-based protein profiling “click chemistry” [51]. Importantly, reducing background florescence is essential for using AdU in tracking proliferative cells.

#### 4.2.2. Isolation of Proliferative Cells for Cell Counting

BrdU labelling is applied extensively for the quantification of proliferating cells *in vitro* and *in vivo*, where direct counts are conducted on microscopy images. Importantly, the near bio-orthagnal reaction for the alkyne-azide conjugation, maximises tissue and cell integrity. Therefore, together with direct cell counting, flow cytometry techniques including Fluorescence Activated Cell Sorting (FACS), may be applied for EdU labelled cells due to the conservation of cell integrity [52]. These techniques have been validated for measuring T-cell proliferation where EdU incorporated into T-lymphocytes were compared to the current gold standard of H^3^-thymidine labelling. When quantified using flow cytometry a near-identical correlation was proposed [52]. The effectiveness of EdU for cell counts in *in vitro* experimentation was compared to BrdU, using both a Neubauer cell counting chamber and flow cytometry in cell cultures [41]. The automated cell counts are facilitated by the ready fluorescence labelling of EdU, together with the maintenance of cell integrity. This in turn enables the molecular profile of cells to be conserved.

#### 4.2.3. The Next Dimension: Proliferating Cells for Molecular Assays

The robust maintenance of cell integrity during EdU labelling will allow EdU labelled cells to be isolated and their molecular profile characterised. Following the micro-dissection and dissociation of cells from culture and tissues, cells incorporating EdU can be fluorescently labelled under conditions maximising the conservation of their RNA content. The cells can be isolated using FACS, the RNA extracted and used to profile the cells using real time PCR or micro-arrays. These techniques are not applicable to BrdU labelled cells as the detection protocols will destroy the molecular content of the cells. Preliminary findings demonstrating the utility of this technique has been reported by Cavanagh *et al*. [53]. Despite these significant advantages of EdU labelling, the requirement of copper ions to catalyse the reaction has limited its application within living cells. 

## 5. EdU for Probing Living Systems

The use of “click chemistry” for tracking DNA synthesis in living cells and animals is hampered by the cytotoxic effects of copper at the concentrations required for catalysing the click reaction [54]. As reviewed by Becer and colleagues [55], “click chemistry” has been used in a variety of fields, with multiple metal-free click reactions being investigated (Table 1) [56]. Based on the observation by Wittig and Krebs that the exothermic cycloaddition of cyclooctyne with phenyl azide produces the corresponding triazole in good yields [57], the Bertozzi group examined strain-promoted azide-alkyne [3+2] cycloaddition of azides and cyclooctyne derivatives [58]. The cyclooctyne derivatives react with the azide without copper as a catalyst due to the geometrical deformation of the alkyne bond arising from ring strain [59]. The cycloaddition kinetics was slower than copper catalysed Huisgen azide-alkyne cycloadditions (CuAAC).

**Table 1:** A scheme summarising the application of Cu-free click chemistry reactions.

Catalysis-free cycloadditions	Structure Example	Application	Reference
Cyclooctyne Derivaties	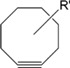	Detection of glycan- associated azides on cell surface	[58]
Difluorinated Cyclooctynes	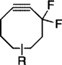	Visualization of cell surface glycan- associated azides / glycan trafficking	[60]
Hydrophilic Azacyclooctyne	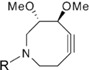	Visualization of glycan- associated azides within cell lysates and on the surface of live cells	[61]
Dibenzocylooctynol Derivatives	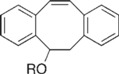	Visualization of cell surface glycan- associated azides / glycan trafficking	[62]
Photo-Triggering of Cyclopropenone	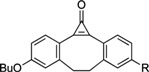	Visualization of cell surface glycan- associated azides / glycan trafficking	[63]
Cyclooctyne-FLAG Conjugate	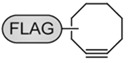	Visualization of cell surface glycan- associated azides in various organs	[64]
Electron-Deficient Alkynes	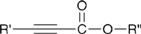	Introducing functional groups to DNA – *in vitro*	[65]

The slow rate of cycloaddition with cyclooctyne derivatives is improved by introducing fluorine as an electron-withdrawing substituent on the α postion of the triple bond [60,66,67]. The difluorinated cyclo-octyne was further improved to be synthetically traceable [68]. In the same year, a novel azacyclo-octyne was reported to have superior polarity and water solubility which in turn reduced nonspecific binding, thereby improving sensitivity of azide detection [61]. Dibenzocylooctynol derivatives also allow for Cu-free “click chemistry” [62]. In the following year, reactive dibenzocyclooctynes were produced by the photo-triggering of a cyclopropenone [63].

This allows for the labelling of living organisms in a temporally and spatially controlled manner. In all studies, the use of cyclo-octyne derivatives have been shown to be non-toxic and compatible in labelling and monitoring cell surface glycoproteins as well as the labelling of lipids in live cell cultures. Recently, Cu-free “click chemistry” using a variety of cyclo-octyne probes was demonstrated to label azidosugars in mice [64]. However, the cyclooctyne derivatives are large complicated reactive groups and this may be disadvantageous in many applications [55].

A synthetic protocol for the 1,3-dipolar cycloaddition of azides with electron-deficient alkynes was reported in 2004 to occur under physiological conditions [65]. The electron-deficient alkyne favours the Huisgen cycloaddition, thus bypassing the need for a catalyst [40]. The study reported the coupling of an azido-DNA (5′-end) molecule with an electron-deficient alkyne, potentially allowing for the introduction of functional groups to DNA. This raises the possibility of copper free “click chemistry” in tracking DNA synthesis in living cells and animals. The translation of applicability of these labelling techniques used in detecting cell surface or structural proteins, to their application in labelling DNA in living cells, must be considered with caution. Together with the known cytotoxicity associated with the incorporation of high concentrations of the unnatural bases into DNA, the additional covalent conjugation of probes to these bases would have a profound impact on DNA stability and transcription, thereby influencing cell function.

### EdU, A Unique Probe

As terminal alkyne groups are rarely present in biological systems, direct detection of the carbon to carbon triple bond would allow for the identification of EdU within DNA. Raman confocal spectroscopy enables the focal detection of specific chemical subunits within cells. Using this technique, an intense alkyne peak at (2,122 cm^−1^) was recently demonstrated in living HeLa cells following EdU incorporation into their replicating DNA [69]. EdU, when incorporated into DNA at lower concentrations, does not impact the biology of cells [48]. Therefore EdU has the potential to track, in living cells, DNA replication and the fate of cells labelled during replication. Whilst there are limitations with Raman spectroscopy, it demonstrates a concept of growing potential, when linked with improving detection techniques.

## 6. Summary

Tracking cell division through incorporating unnatural bases into newly synthesised DNA is a powerful and widely used tool in biology. Initial difficulties detecting tritiated thymidine was largely overcome using its halogenated analogues. The necessity for the immunodetection of these halogenated probes constrained studies, limiting them largely to anatomical investigations. The recent emergence of EdU, detected at high efficiency at near bio-orthogonal conditions, using selective “click chemistry”, allows investigation of DNA synthesis and cell genesis in a variety of assays. These have been extended to include living cells. These advances in tracking DNA synthesis are complemented by parallel advances in anatomical and molecular biology techniques. These include increasing sensitivity and automation of assays and detection technologies. Thus our capacity to investigate the most fundamental and secure process of life, DNA synthesis *per-se* is increased, facilitating the study of the replication and passage of life. The full potential of EdU and related molecules in biology and medicine, largely remain to be elucidated.
